# Do Knee Pain Phenotypes Have Different Risks of Total Knee Replacement?

**DOI:** 10.3390/jcm9030632

**Published:** 2020-02-27

**Authors:** Feng Pan, Jing Tian, Ishanka P. Munugoda, Stephen Graves, Michelle Lorimer, Flavia Cicuttini, Graeme Jones

**Affiliations:** 1 Menzies Institute for Medical Research, University of Tasmania, Private Bag 23, Hobart, Tasmania 7000, Australia; j.tian@utas.edu.au (J.T.); ishanka.munugoda@utas.edu.au (I.P.M.); graeme.jones@utas.edu.au (G.J.); 2 Australian Orthopaedic Association National Joint Replacement Registry (AOANJRR), Adelaide, South Australia 5000, Australia; segraves@aoanjrr.org.au; 3 South Australian Health and Medical Research Institute (SAHMRI), Adelaide, South Australia 5000, Australia; michelle.lorimer@sahmri.com; 4 Department of Epidemiology and Preventive Medicine, Monash University Medical School, Commercial Road, Melbourne, VIC 3181, Australia; flavia.cicuttini@monash.edu

**Keywords:** knee pain, pain subgroup, phenotype, knee replacement, risk

## Abstract

Pain is the main impetus for osteoarthritis (OA) patients to seek healthcare including joint replacement. The pain experience in OA is heterogeneous and affected by factors across multiple domains—peripheral, psychological, and neurological. This indicates the existence of homogenous subgroups/phenotypes within OA patients with pain. We recently identified three pain phenotypes using a wide spectrum of pain-related factors, including structural damage on magnetic resonance imaging (MRI), emotional problems, number of painful sites, sex, body mass index, education level and comorbidities (i.e., Class 1: high prevalence of emotional problems and low prevalence of structural damage (25%); Class 2: low prevalence of emotional problems and high prevalence of structural damage (20%); Class 3: low prevalence of emotional problems and low prevalence of structural damage (55%)). This study was to examine whether the total knee replacement (TKR) risk over 12 years was different among these three pain phenotypes. Data on 963 participants (mean age 62.8 ± 7.4 years) from a population-based cohort study were utilised. Data on socio-demographic, psychological and comorbidities were collected. MRI of the right knee structural pathology was performed. TKR history was ascertained by linking to the Australian Orthopedic Association National Joint Replacement Registry. Latent class analysis and the Cox proportional hazards model were applied for the analysis. During the follow-up period, 41 right and 44 left TKRs in 67 participants were identified. In multivariable analyses, participants in Class 1 and 2 had a higher risk of having a TKR (Class 1 vs. Class 3, HR (hazard ratio) 4.81, 95%CI (confidence interval) 2.33–9.93; Class 2 vs. Class 3, HR 9.23, 95%CI 4.66–18.30). These associations were stronger in the imaged right knee but were also significant in the left knee. Participants within distinct pain phenotypes have different risks of TKR, suggesting that the identified phenotypes reflect distinct clinical subgroups with different prognoses. The risk for TKR was higher in Class 1 than that in Class 3, suggesting that pain/emotional status is a stronger driver for TKR than structural damage, and that selecting patients for TKR needs to be optimized in clinical practice.

## 1. Introduction

Osteoarthritis (OA) is a heterogeneous and progressive disease, and is a major cause of pain and decreased function in the aging population [1]. The knee is the most commonly affected site, being ranked as the 11th highest contributor (with hip OA) of the 291 conditions to global disability [2]. There is no cure, and existing treatments primarily focusing on alleviating pain are unsatisfactory. Total knee replacement (TKR) is effective to reduce pain and improve functioning for a majority of patients with end-stage OA who have failed to respond to pharmacological and conservative treatments [3]; however, up to 34% patients who undergo surgery still experience ongoing pain [4]. In Australia, there were more than 50,000 TKRs performed to treat OA in 2017 [5], at an estimated cost of AU$19,000 to AU$30,000 per patient [6]. In United States (US), more than 640,000 knee replacement procedures are undertaken annually costing about US$10.2 billion [3]. OA thus poses a large individual as well as societal and economic burden related to management of the disease.

OA is considered not one disease, but consists of several subgroups/phenotypes, leading to a large variation in clinical presentations (e.g., pain) and response to OA treatments [7,8]. It has been suggested that each subgroup/phenotype differs in underlying causes and mechanisms, and thus requires different treatments [8]. Suboptimal patient outcomes and lack of treatment efficacy in some patients may be attributed to the current ‘one-size fits all’ approach which fails to select the optimal patient. There have been some attempts to characterise distinct knee OA phenotypes by grouping a collection of patients’ observable characteristics or traits [7,9]. However, the cross-sectional design in most previous studies limits the validity of this phenotype and its clinical relevance.

Pain is the most defining symptom of OA. The pain experience is complex and affected by multiple factors. It has been suggested that the three most important domains are peripheral, psychological and neurological factors as they are modifiable through interventions [10]. Studies in an attempt to identify pain phenotypes in OA have been cross-sectional and defined unidimensional phenotypes [11,12,13,14,15]. To fill in this gap, we recently performed a study to identify pain phenotypes by considering a wide spectrum of factors, including magnetic resonance imaging (MRI)-detected structural lesions, body mass index (BMI), comorbidities and psychological factors, etc. [16]. This study identified three distinct pain phenotypes (i.e., Class 1: a high prevalence of emotional problems and low prevalence of structural damage; Class 2: a high prevalence of structural damage and low prevalence of emotional problems; Class 3: a low prevalence of emotional problems and low prevalence of structural damage). Those in Class 1 had a greater pain severity and number of painful sites than those in Class 2 and 3 at each time-point over a mean follow-up of 10.7 years. This suggests that the phenotypes reflect distinct clinical syndromes. Given that pain is the most important factor for the physician’s decision to make a referral for joint replacement [17], we hypothesised that the risk for having a TKR differs across these three phenotypes. Therefore, to validate these phenotypes being clinically relevant for TKR, we sought to examine whether distinct phenotypes have a different risk for TKR by linking to the Australian Orthopaedic Association National Joint Replacement Registry (AOANJRR) over a mean follow-up of 12.0 years.

## 2. Materials and Methods

### 2.1. Participants

The data obtained for the analysis of this study were from the Tasmanian Older Adult Cohort (TASOAC) Study. The TASOAC study is a longitudinal and population-based cohort study, consisting of participants aged 50–80 years who were randomly selected from the electoral roll (population *n* = 229,000) in 2002–2004 in Southern Tasmania, Australia. A total of 1099 participants were recruited at baseline, and 875, 768 and 563 participants were tracked with follow-up assessments at a mean follow-up period of 2.6, 5.1 and 10.7 years, respectively. The assessments included general questionnaires and interview (e.g., risk factors, pain, medical history) and clinical assessments. Ethics was approved by the Southern Tasmanian Health and Medical Human Research Ethics Committee. All participants provided informed consent.

### 2.2. Measurements for Factors to Identify Pain Phenotypes

#### 2.2.1. Knee Structural Abnormalities on MRI 

Each participant had an MRI scan on their right knee in the sagittal plane on a 1.5-T whole body MR unit (Picker, OH) using a commercial transmit–receive extremity coil. The sequences used have been previously described [18]. Cartilage defects were assessed on a scale 0–4 by a trained observer on T1-weighted MR images at the medial tibial, medial femoral, lateral tibial and lateral femoral sites, as previously described [19]. Intraobserver repeatability (using intraclass correlation coefficients, ICCs) ranged from 0.80 to 0.95. Interobserver reliability ranged from 0.89 to 0.94. A score of 2 or greater at any site was considered the presence of cartilage defect [20,21]. Bone marrow lesions (BMLs) were graded on a 0–3 scale by a trained observer on T2-weighted MR images in the subregion of medial tibial, medial femoral, lateral tibial and lateral femoral sites and defined as areas of increased signal adjacent to the subcortical bone, as previously described [22]. ICCs for intraobserver repeatability were 0.89–1.00. The presence of BML was defined as a score of 1 or greater at any site [23]. As previously described [24], effusion-synovitis was scored on a 0–3 scale by a trained observer on T2–weighted MR images in the suprapatellar pouch. The intraclass reliability assessed as weighted *κ* was 0.63–0.75, and the interclass inter-rater reliability was 0.65–0.79. The presence of effusion-synovitis was defined as a score of 2 or greater [25].

#### 2.2.2. Emotional Problems

Emotional problems were assessed by using one single mental health item from the short form-8. The participants were asked ‘how much have you been bothered by emotional problems during the past four weeks, such as feeling anxious, depressed or irritable?’. Responses included ‘not at all’, ‘very little’, ‘moderately’, ‘quite a lot’ and ‘extremely’. The presence of emotional problems was defined as a response of ‘very little’ or more.

#### 2.2.3. Number of Painful Sites

Participants reported whether they had pain (yes/no) occurring at their neck, back, hands, shoulders, hips, knees or feet. A total number of painful sites was created by summing each site (ranging 0–7).

#### 2.2.4. Other Pain-Related Factors

Weight and height were measured, then BMI was calculated (kg/m^2^). Sex was collected. Highest education level participants had completed was self-reported and grouped into three categories: low = school only, medium = trade/vocational certificate, high = university level or above. A self-reported comorbidity questionnaire was used to record participant’s common conditions. Four of the most common conditions (i.e., heart attack, hypertension, diabetes and rheumatoid arthritis) in this population were extracted. The presence of comorbidity was defined as participants having any of these four comorbidities.

### 2.3. Measurement for Outcome

#### Total Knee Replacement

The AOANJRR collects data for individuals receiving joint replacement surgery at both public and private hospitals in Australia. The data collection in Tasmania started from September 2000. First-time TKR of participants in this study was determined by data linkage to AOANJRR. We excluded participants who had a TKR prior to their baseline visit, therefore, TKR undertaken between 1 March 2002 and 21 September 2016 were extracted. Using a sequential multi-level matching process, we validated the data against State and Territory Health Department data. The following data were obtained, including the procedure date, side of TKR, and the reason for the procedure (e.g., OA). Procedures only performed for OA were considered in this study.

### 2.4. Measurements for Other Related Factors

Age was asked. Radiograph of the right knee was performed and graded on a scale 0–3 according to the Altman atlas for osteophytes and joint space narrowing (JSN), as previously described [26]. ICCs were 0.98–0.99 for intraobserver repeatability. The presence of knee radiographic OA (ROA) was defined as any score of 1 or greater for JSN or osteophytes [27].

### 2.5. Statistical Analysis

As previously described [16], latent class analysis (LCA) was applied to identify underlying subgroups with similar profiles based on multiple observed individual characteristics, pain-related risk factors (i.e., knee structural damage on MRI, emotional problems, number of painful sites, sex, BMI, education level and comorbidities) were included into the LCA model. To determine an optimal model, we tested models with two-six classes and then compared the fit of each model. The following criteria were considered in the model selection: (1) the Bayesian information criterion (BIC), Aikaike’s information criterion (AIC), consistent AIC (cAIC) and log-likelihood (LL) [28]. Lower AIC and BIC values and larger LL (absolute value) indicate a better model fit; (2) The posterior probabilities (PPs) are the probability of the observations that are classified in a given class. A greater PP indicates an optimised number of classes; (3) Distinctiveness of class and clinical relevance.

The association between identified pain phenotypes and TKR was estimated by using the Cox proportional hazards model where an estimate of the hazard ratio (HR) with 95% confidence interval (CI) was provided. The participants were censored at the date of death or the date they left Australia or 21 September 2016 (the end of the observation period) if they do not experience a TKR. As of the censoring date, a participant who dies or leaves Australia permanently was considered a non-event participant. A Kaplan–Meier curve was used to depict the time until study participants had their first TKR.

Stata V.15 was used for the analyses, and LCA analysis was performed by using LCA Stata Plugin [29]. *p* values less than 0.05 (two-tailed) were regarded as statistically significant.

## 3. Results

### 3.1. Participants Included for Identifying Pain Phenotypes

**Figure 1:**
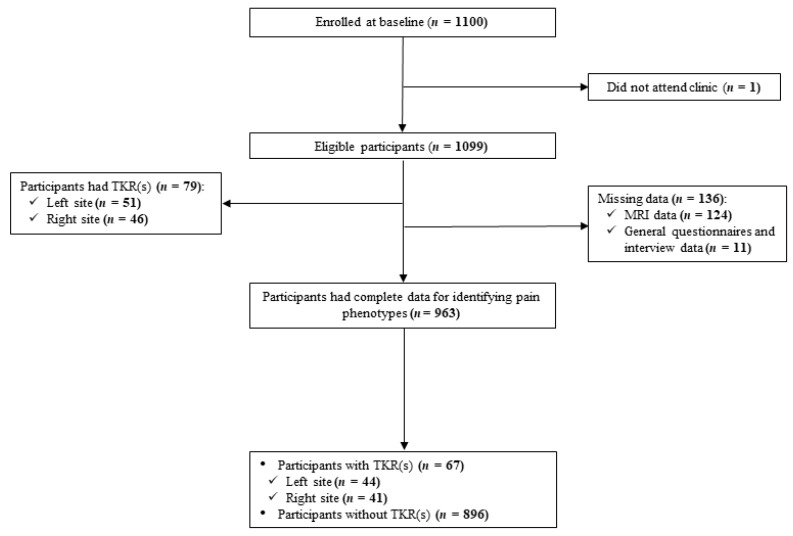
Flow chart of participants included in this study.

There were 1099 participants who attended the baseline assessments. As shown in Figure 1, 136 participants were excluded due to missing MRI and general questionnaire or interview data, therefore, LCA analysis included data from 963 participants. The mean age and BMI were 62.8 years (SD 7.4) and 27.7 kg/m^2^, 50% of participants were female.

### 3.2. Participants’ Characteristics across Pain Phenotypes

According to the criteria for the model selection, the 3-Class was considered an optimal model as compared to 2-Class and 4 to 6-Class. A collection of fit indices is shown in the Supplementary Table S1. Class 1 and 2 comprised 25% (*n* = 236) and 20% (*n* = 193) of the sample, respectively, while the majority of participants were assigned to the Class 3 (55%, *n* = 534). Participants’ characteristics by three classes are displayed in Table 1. Participants’ characteristics were different across three phenotypes in terms of MRI-detected structural damage, socio-demographic factors as well as pain intensity and number of painful sites. Among these factors, the most substantial difference was the proportion of participants having MRI-detected structural damage and emotional problems. Namely, in Class 1, 92% of participants had emotional problems and 35%–43% of participants had a different type of structural damage. In Class 2, half of the participants reported the presence of emotional problems and as high as 89% had at least one structural damage. The proportion of participants in Class 3 having emotional problems was similar to those in Class 2, while they had a relatively low prevalence of structural damage.

### 3.3. First-Time TKR Due to OA

**Figure 2:**
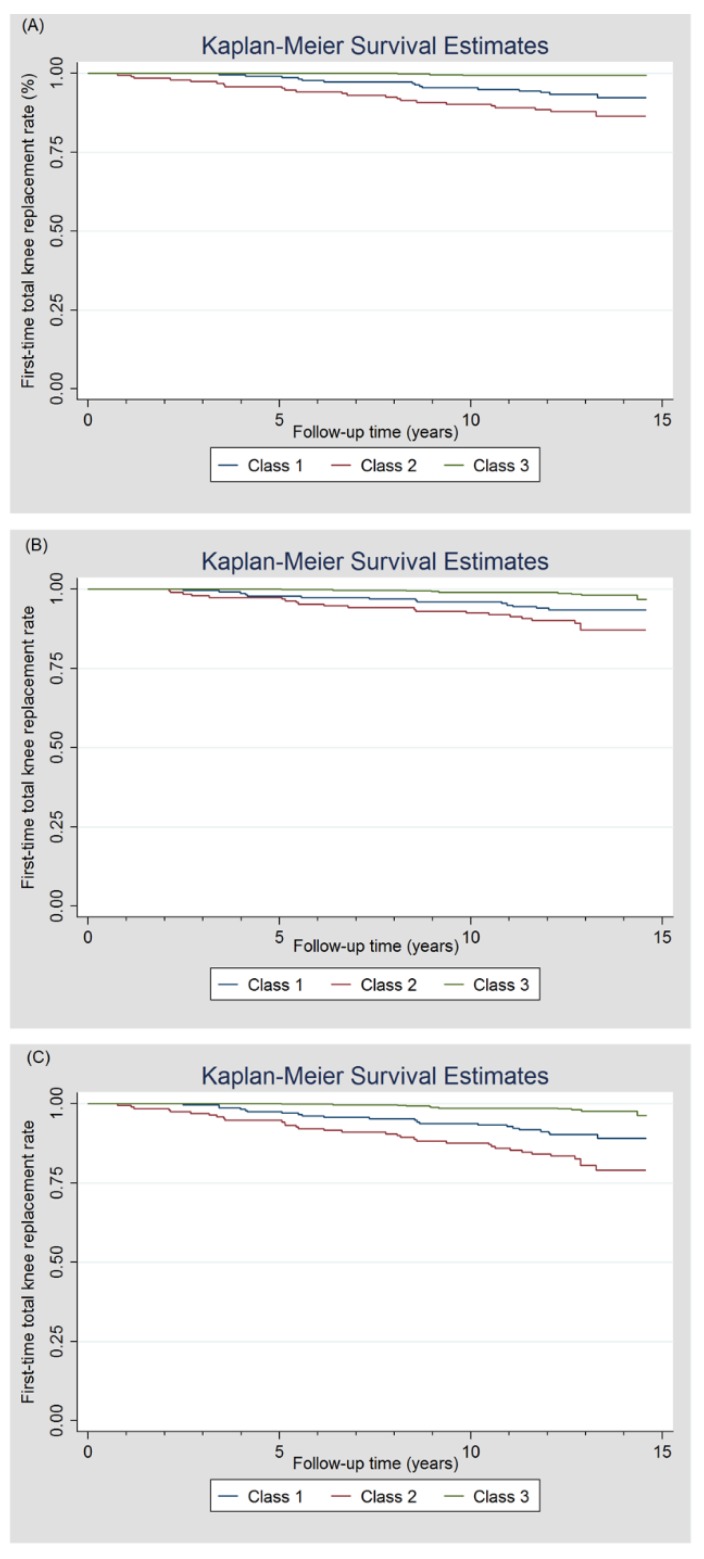
The first-time total knee replacement rate was plotted by a Kaplan–Meier curve over a mean observation period of 12.0 years. (**A**) right knee; (**B**) left knee; (**C**) any knee.

Among a total of 1099 participants, 46 right and 51 left TKRs in 79 participants were identified during a mean observation time of 12.0 years (Figure 1). Of 963 participants included in the LCA analysis, 67 participants had a TKR either at right (*n* = 41) and/or left (*n* = 44) knee. Table 2 shows the details of first-time TKR in each class. Class 2 had a higher rate of people with a TKR in right, left or any knee compared to Class 1 and Class 3. The time to first-time TKR by pain phenotypes was analysed by a Kaplan–Meier curve (Figure 2). The first-time TKR rate in right, left and any knee in participants in Class 2 was higher and increased faster relative to those in Class 1 and Class 3. Site-specific associations between pain phenotypes and TKR, estimated by using the Cox proportional hazards model, are presented in Table 3. In Class 2 and Class 1, risk estimates of first-time TKR were 9.59 and 4.87-fold higher in comparison with Class 3 in univariable analysis. Also, HR was greater in Class 2 than that in Class 1. These associations remained statistically significant after adjustment for age. We observed a stronger association between pain phenotypes and TKR in the right knee where the MRI-detected structural pathology came from than left knee.

## 4. Discussion

In the present study, we estimated the rate of having a TKR among three pain phenotypes we previously identified. We found that both Class 1 and 2 had a higher TKR rate than Class 3, and the rate of TKR in Class 2 was highest among the three classes. While the association with Class 2 was expected, the numerically weaker but also strong association with Class 1 was unexpected, suggesting that pain is a stronger driver of having a TKR than structural change. By assessing the associations between pain phenotypes and distal outcome (TKR), the findings not only provide evidence of differences in clinical prognoses in distinct pain phenotypes, but also phenotypes may be of clinical relevance to selecting and optimising patients’ classification for future treatments. To the best of our knowledge, this study, for the first time, evaluates the association between pain phenotypes and the risk of TKR.

Phenotyping OA has attracted interest due to the potential to develop tailored therapies for a selected subgroup of patients. Two recent systematic reviews concluded that knee OA is not one disease, but consists of different phenotypes [7,9]. OA pain is a complex experience determined by multiple factors such as peripheral structural, psychological and neurological etc. These risk factors are not commonly shared with OA pathology, indicating that the mechanisms underlying OA pathology and pain are different, and thus OA and pain might be different clinical entities. To date, studies to identify OA pain phenotypes are scarce [11,12,13,14,15], most of which are limited to a single dimension pain phenotypes and/or cross-sectional study design. This precludes identification of multi-dimension pain phenotype and validation of the construct of pain phenotype and its clinical relevance [9].

There was only one study by Kittelson et al. [15] that considered multiple pain dimensions aimed at identifying pain phenotypes in OA. They identified four pain phenotypes (i.e., higher levels of comorbidities; higher knee joint sensitivity; higher levels of psychological distress; and minimal joint disease and pain). Despite differences in number of classes between studies, our study finding of two pain phenotypes (i.e., Class 1 and 3) is similar to the phenotypes identified in study by Kittelson et al. (i.e., higher levels of psychological distress and minimal joint disease and pain). The phenotypes are not directly comparable between studies due to the differences in participants’ characteristics and variables included in the LCA analysis. A significant difference in psychological and structural factors underscores their important contributory roles in pain genesis and reinforces the inclusion of them in determining pain phenotypes.

There have been no studies directly investigating the impact of psychological factors on TKR, but prior studies primarily focused on influence of psychological factors on pain and function after TKR. Preoperative psychological factors have been identified as important risk factors for poor outcome in patients undergoing TKR [30,31]. A recent systematic review including 17 cohort studies concluded that psychological factors are predictive of poor clinical outcomes including patients’ satisfaction, pain or function after TKR [32]. This is concerning given our findings that participants with a high prevalence of emotional problems in Class 1 had a higher risk of having a TKR compared to Class 3. However, the reasons for an inappropriately high rate of TKR in participants in Class 1 remain to be elucidated given the fact that the indications for undergoing a TKR are largely based on symptoms (pain) usually in combination with pathology. Furthermore, it has been suggested that 10%–34% of patients had persistent pain after knee joint replacement [4], this proportion is comparable to the percentage of participants classified in Class 1 (25%). In light of this, it would appear that there is a scope for improvement in how orthopedic surgeons determine whether a patient is appropriate for TKR at an individual level.

We previously reported that Class 1 had a higher pain severity than Class 2 and 3 at each time-point and these pain phenotypes remained stable over time [16]. Given that pain is the most important factor in making a decision for TKR [4], we hypothesized that participants in Class 1 may have a higher risk of taking a TKR than those in Class 2 and 3. Instead, Class 2 had the highest risk. This may reflect the complexity of the TKR decision-making process in clinical care where the individual decisions of both patient and surgeon have a role in determining a TKR [17,33]. It is possible that pain generated in Class 2 is mainly driven by peripheral structural damage, therefore, participants in Class 2 were more likely to benefit from TKR than those in Class 1. There was a total of 12 TKRs conducted in either knee among participants in Class 3 where a low prevalence of structural damage and emotional problems predominate. One possible explanation is that the disease may progress quickly in some patients during the long follow-up period.

Long follow-up period, TKR confirmed via linkage to the National Joint Replacement Registry and multi-dimension pain phenotypes are the strengths of this study. There are several limitations in the current study. First, this study aimed to include all factors related to pain in the identification of phenotypes; however, some other important factors (e.g., inflammatory factors) are only available in a subsample. In addition, most of the variables (e.g., MRI-detected structural damage) included in the LCA analysis were not assessed at all follow-ups, precluding our ability to evaluate the stability of pain phenotypes. However, we previously reported that Class 1 had a higher pain severity and greater number of painful sites than Class 2 and 3 at each time-point, providing preliminary evidence that pain phenotypes appear stable over time [16]. Second, MRI-detected structural damage was only measured at the right knee, therefore, phenotypes may be more specific and accurate in predicting TKR at the right knee. This may have led to an underestimation of the association between pain phenotypes and TKR at left or any knees. Supporting this, we found a greater effect size at the right knee than that of left knee.

In conclusion, participants in distinct pain phenotype groups have different risks of TKR. This suggests that the identified phenotypes reflect distinct clinical subgroups with different prognoses. However, those participants with low structural damage have an increased risk of TKR when they have high compared to low emotional problems, indicating that pain/emotional status is a more important risk factor for TKR than structural damage. Selection of patients for TKR could be optimised with integration of mental health professionals by screening out those in Class 1.

## Figures and Tables

**Table 1:** Participants’ characteristics at baseline by pain phenotypes *.

Characteristics	Class 1 (*n* = 236)	Class 2 (*n* = 193)	Class 3 (*n* = 534)	*p*^†^ C1 vs. C2	*p*^†^ C1 vs. C3	*p*^†^ C2 vs. C3
Age (years)	63.1 ± 7.7	64.4 ± 7.4	62.1 ± 7.2	0.217	0.242	0.001
Female sex, *n* (%)	152 (64)	66 (34)	267 (50)	<0.001	<0.001	<0.001
BMI (kg/m^2^)	29.0 ± 5.4	29.4 ± 4.5	26.5 ± 3.9	0.869	<0.001	<0.001
Presence of emotional problems, *n* (%)	218 (92)	97 (50)	298 (56)	<0.001	<0.001	0.040
Emotional problems, *n* (%)				<0.001	<0.001	0.003
Not at all	18 (8)	96 (50)	236 (44)			
Very little	94 (40)	69 (36)	185 (35)			
Moderately	86 (36)	22 (11)	84 (16)			
Quite a lot	35 (14)	5 (3)	27 (5)			
Extremely	4 (2)	1 (1)	2 (0.4)			
Education level, *n* (%)				<0.001	<0.001	<0.001
School only	160 (68)	77 (40)	296 (55)			
Vocation training	74 (31)	77 (40)	164 (31)			
University or higher	2 (1)	39 (20)	74 (14)			
Presence of any comorbidity, *n* (%)	162 (69)	98 (51)	198 (37)	<0.001	<0.001	<0.001
WOMAC pain score (0–45)	8.6 ± 8.6	3.2 ± 5.6	1.5 ± 3.0	<0.001	<0.001	0.001
Number of painful sites (0–7)	5.8 ± 1.1	2.4 ± 1.5	2.2 ± 1.7	<0.001	<0.001	0.265
Presence of radiographic knee OA, *n* (%)	156 (66)	131 (67)	290 (54)	0.791	0.004	0.004
Knee structural pathology, *n* (%)						
Cartilage defects	88 (37)	172 (89)	60 (11)	<0.001	<0.001	<0.001
BMLs	82 (35)	161 (83)	101 (19)	<0.001	<0.001	<0.001
Effusion-synovitis	102 (43)	125 (65)	184 (34)	<0.001	0.026	<0.001

* Values are the Mean ± SD except for percentages and pain phenotypes (Classes) were identified from latent class analysis. ^†^ *p* values are from post hoc testing for comparisons between classes determined by ANOVA or logistic regression (where appropriate); BMI body mass index; WOMAC Western Ontario and McMaster Universities Osteoarthritis Index; OA osteoarthritis; BMLs bone marrow lesions. This table has been published elsewhere [16] and permission to use has been obtained.

**Table 2:** Details of first-time total knee replacement across three pain phenotypes.

	Class 1 (*n* = 236)	Class 2 (*n* = 193)	Class 3 (*n* = 534)	*p* * C1 vs. C2	*p* * C1 vs. C3	*p* * C2 vs. C3
Mean time to first-time TKR, years						
Right knee	8.2 ± 3.1	6.4 ± 3.8	8.9 ± 0.9	0.385	1.000	0.722
Left knee	7.5 ± 3.8	7.4 ± 3.8	10.0 ± 3.2	1.000	0.328	0.248
Any knee	7.7 ± 3.5	7.1 ± 3.9	9.7 ± 2.9	1.000	0.419	0.133
Number of TKR, *n* (%)						
Right knee	15 (6)	23 (12)	3 (1)	0.047	<0.001	<0.001
Left knee	14 (6)	21 (11)	9 (2)	0.066	0.003	<0.001
Any knee	22 (9)	34 (18)	11 (2)	0.012	<0.001	<0.001

* *p* values are from post hoc testing for comparisons between classes determined by ANOVA; TKR total knee replacement.

**Table 3:** Identified pain phenotypes and knee joint replacement.

	Class 1 vs. Class 3		Class 2 vs. Class 3		Class 2 vs. Class 1	
	Univariable HR(95%CI)	Multivariable * HR(95%CI)	Univariable HR(95%CI)	Multivariable * HR(95%CI)	Univariable HR(95%CI)	Multivariable * HR(95%CI)
Any	**4.87 (2.36, 10.05)**	**4.81 (2.33, 9.93)**	**9.59 (4.86, 18.93)**	**9.23 (4.66, 18.30)**	**1.97 (1.15, 3.37)**	**1.92 (1.12, 3.29)**
Right	**11.93 (3.45, 41.21)**	**11.96 (3.46, 41.34)**	**23.07 (6.93, 76.88)**	**23.26 (6.95, 77.77)**	**1.93 (1.01, 3.71)**	**1.94 (1.01, 3.74)**
Left	**3.72 (1.61, 8.62)**	**3.63 (1.57, 8.40)**	**6.90 (3.16, 15.08)**	**6.42 (2.93, 14.09)**	1.85 (0.94, 3.64)	1.77 (0.90, 3.48)

Bold denotes statistically significant result; HR, hazard ratio; CI, confidence interval; * Adjusted for age.

## Supplementary Materials

The following are available online at https://www.mdpi.com/2077-0383/9/3/632/s1, Table S1: Goodness of fit indices and posterior probabilities of latent class analysis
