# Supplementary material for: Do Knee Pain Phenotypes Have Different Risks of Total Knee Replacement?
Source: J Clin Med. 2020 Feb 27;9(3):632. doi: 10.3390/jcm9030632 (PMC7141124; doi:10.3390/jcm9030632)
Supplement: Supplementary file 1 [file jcm-09-00632-s001.pdf]

Journal of
Clinical Medicine

MDPI

Supplementary

Table 1. Goodness of fit indices and posterior probabilities of latent class analysis.

#Latent classes	AIC	BIC	Consistent AIC	Log-likelihood	Mean posterior probabilities
**2**	3484	3699	3743	-8931	0.79/0.88
**3**	3374	3698	3764	-8854	0.75/0.79/0.82
**4**	3335	3770	3857	-8812	0.68/0.78/0.78/0.80
**5**	3296	3841	3950	-8771	0.71/0.72/0.75/0.77/0.80
**6**	3280	3935	4066	-8741	0.70/0.71/0.73/0.74/0.80/0.83
